# Alterations of Gut Microbiota in Patients With Graves’ Disease

**DOI:** 10.3389/fcimb.2021.663131

**Published:** 2021-05-05

**Authors:** Shih-Cheng Chang, Shu-Fu Lin, Szu-Tah Chen, Pi-Yueh Chang, Yuan-Ming Yeh, Fu-Sung Lo, Jang-Jih Lu

**Affiliations:** ^1^ Department of Laboratory Medicine, Linkou Chang Gung Memorial Hospital, Taoyuan, Taiwan; ^2^ Department of Medical Biotechnology and Laboratory Science, Chang Gung University, Taoyuan, Taiwan; ^3^ Division of Endocrinology and Metabolism, Department of Internal Medicine, Linkou Chang Gung Memorial Hospital, Taoyuan, Taiwan; ^4^ College of Medicine, Chang Gung University, Taoyuan, Taiwan; ^5^ Genomic Medicine Core Laboratory, Linkou Chang Gung Memorial Hospital, Taoyuan, Taiwan; ^6^ Department of Pediatrics, Division of Pediatric Endocrinology, Linkou Chang Gung Memorial Hospital, Taoyuan, Taiwan

**Keywords:** Graves’ disease, gut microbiota, clinical parameters, 16S rRNA, next-generation sequencing

## Abstract

Graves’ disease (GD) is a systemic autoimmune disease characterized by hyperthyroidism. Evidence suggests that alterations to the gut microbiota may be involved in the development of autoimmune disorders. The aim of this study was to characterize the composition of gut microbiota in GD patients. Fecal samples were collected from 55 GD patients and 48 healthy controls. Using 16S rRNA gene amplification and sequencing, the overall bacterial richness and diversity were found to be similar between GD patients and healthy controls. However, principal coordinate analysis and partial least squares-discriminant analysis showed that the overall gut microbiota composition was significantly different (ANOSIM; p < 0.001). The linear discriminant analysis effect size revealed that *Firmicutes* phylum decreased in GD patients, with a corresponding increase in *Bacteroidetes* phylum compared to healthy controls. In addition, the families *Prevotellaceae*, and *Veillonellaceae* and the genus *Prevotella_9* were closely associated with GD patients, while the families *Lachnospiraceae* and *Ruminococcaceae* and the genera *Faecalibacterium*, *Lachnospira*, and *Lachnospiraceae NK4A136* were associated with healthy controls. Metagenomic profiles analysis yielded 22 statistically significant bacterial taxa: 18 taxa were increased and 4 taxa were decreased. Key bacterial taxa with different abundances between the two groups were strongly correlated with GD-associated clinical parameters using Spearman’s correlation analysis. Importantly, the discriminant model based on predominant microbiota could effectively distinguish GD patients from healthy controls (AUC = 0.825). Thus, the gut microbiota composition between GD patients and healthy controls is significantly difference, indicating that gut microbiota may play a role in the pathogenesis of GD. Further studies are needed to fully elucidate the role of gut microbiota in the development of GD.

## Introduction

Graves’ disease (GD) is an autoimmune disorder in which stimulatory autoantibodies activate thyroid stimulating hormone receptors (TSHR), leading to excessive amounts of thyroid hormones. It is the most common cause of hyperthyroidism, with typical symptoms including weight loss, fatigue, heat intolerance, tremor, and palpitations. GD can occur at all ages, but is more common in women than in men (Ehlers et al., 2019). The prevalence of hyperthyroidism in the United States is approximately 1.2% (0.5% overt and 0.7% subclinical) (Doubleday and Sippel, 2020). The prevalence rate of overt hyperthyroidism estimated by a population-based study in Taiwan is around 0.27-0.37%, with an annual incidence of 0.97 to 1.06 cases per 1,000 individuals (Kornelius et al., 2018). The pathogenesis of GD remains unclear, but it is hypothesized that infections, iodine intake, smoking, and mental stress play a role, and both genetic and environmental factors appear to be involved (Shukla et al., 2018).

The human gut microbiota harbors up to 100 trillion microbes. These microbes play a crucial role in the maintenance of human health by affecting nutrient digestion, immune response, metabolic homeostasis, and protection against pathogen colonization (Lin and Zhang, 2017; Pickard et al., 2017). An imbalance in the gut microbiota or dysbiosis can increase epithelial barrier dysfunction and lead to intestinal and systemic disorders. Recent studies have established a link between the alterations in the composition of gut microbiota in many disease states, including inflammatory bowel disease, Crohn’s disease, obesity, and type 2 diabetes (Ortega et al., 2020; Szilagyi, 2020). In addition, the components and metabolites derived from the gut microbiota are key regulators of host immune responses. Alterations in the gut microbiota may result in an imbalance of T-cell subpopulations, triggering the release of pro-inflammatory and anti-inflammatory cytokines (Lee and Kim, 2017). Moreover, this can trigger the onset of several types of autoimmune diseases, including type 1 diabetes, rheumatoid arthritis, and systemic lupus erythematosus (Alkanani et al., 2015; Correa et al., 2017; Picchianti-Diamanti et al., 2018).

The thyroid gland is the primary endocrine gland in the human body. However, the relationship between the gut microbiota and GD remains unclear. The aim of this study was to characterize the changes in the gut microbiota of patients with GD using 16S rRNA gene sequencing, compare it with the gut microbiota of their healthy counterparts, and determine its possible correlation with the relevant clinical factors.

## Materials and Methods

### Ethics Statement

This study’s protocol was approved by the Institutional Review Board and Ethics Committee of Chang Gung Memorial Hospital (IRB 201701806B0C502). Written informed consent was obtained from all participants before the start of the study. All experimental procedures were performed in accordance with the approved guidelines.

### Study Population and Sample Collection

A total of 55 GD patients were recruited from the Division of Endocrinology and Metabolism at Chang Gung Memorial Hospital between October 2017 and March 2020. These patients were previously diagnosed with GD and had an average follow-up of 45.33 months. All patients were initially treated with anti-thyroid drugs, including propylthiouracil (PTU), methimazole (MMI), or carbimazole (CBZ). GD was diagnosed on the basis of clinical presentations and laboratory data, including symptoms and signs of thyrotoxicosis, diffuse goiter, the presence of ophthalmopathy, elevated free T4 and suppressed TSH levels, and the presence of autoantibody against TSH receptor. In addition, 48 age-, sex-, and BMI-matched healthy subjects from a health screening center constituted the control group. The control group had normal values of free T4, TSH, and thyroid peroxidase (TPO) antibody, and no history of thyroid disease. Free T4 and TSH levels were measured using a one-step chemiluminescent immunoassay (CLIA) on the ADVIA Centaur XP system (Siemens Healthcare, Munich, Germany). TPO antibody and TSH receptor antibody were measured by chemiluminescent microparticle immunoassay (CMIA) (Architect, Abbott, Wiesbaden, Germany). Reference intervals were defined as follows: free T4 0.70-1.48 ng/dL; TSH 0.35-5.50 μIU/mL; anti-TPO <5.61 IU/mL; anti-TSHR <1.75. A questionnaire was used to assess dietary habits and health condition of all subjects. In each group, subjects who met any of the following criteria were excluded from this study: pregnancy; gastrointestinal disorders (chronic diarrhea or inflammatory bowel disease); gout; stroke; cancer; autoimmune diseases (type 1 diabetes, systemic lupus erythematosus, or rheumatoid arthritis); history of gastrointestinal surgery; use of antibiotics, probiotics, prebiotics, symbiotics (<2 months), hormonal medication or Chinese herbal medicine (<3 months); and pure vegetarian. The fecal samples were collected in a clean container, aliquoted, and all aliquots were immediately frozen and stored at ‒80°C.

### 16S rRNA Gene Sequencing

DNA was extracted using the QIAamp Fast DNA Stool Mini Kit (Qiagen, Germany) following the manufacturer’s protocol. Amplification of the 16S V3-V4 region was performed using the universal primers reported previously (Klindworth et al., 2013). Samples were amplified using 2× KAPA HiFi HotStart Ready Mix (KAPA Biosystems, MA, USA), and indexed using the Nextera XT 96 Index Kit (Illumina, CA, USA). Lastly, the library was sequenced on an Illumina MiSeq platform and 300 bp paired-end reads were generated.

### Bioinformatics and Statistical Analysis

The overlapping and merging of paired-end reads were conducted in FLASH (v1.2.11) (Magoc and Salzberg, 2011). Filtering of the raw tags was performed under specific filtering conditions according to the QIIME (v1.9.1) (Caporaso et al., 2010). Chimeras were removed by aligning sequences to the “gold” reference database using UCHIME (Edgar et al., 2011). The resulting filtered sequences were clustered into operational taxonomic units (OTUs) using UPARSE (v7.0.1090) with a 97% similarity threshold. The representative sequences were then classified taxonomically using the ribosomal database project (rdp) classifier (v2.2) with default 0.8 as the confidence threshold. The OTUs were aligned using PyNAST (v1.2) and assigned to taxonomic groups against the Greengenes database (v13.8) or SIlVA database (v132) (Quast et al., 2013). The alpha diversity metrics were estimated in QIIME (v1.9.1), including the coverage percentage (Good’s), Venn diagram, richness estimators (ACE and Chao1), and diversity indices (Shannon and Simpson). Rank‐abundance and rarefaction curves were calculated at a level of 97% similarity of the OTUs. Beta diversity measures, such as principal coordinate analysis (PCoA) and partial least squares-discriminant analysis (PLS-DA), were calculated using QIIME (v1.9.1). The bacterial taxonomic distributions of the sample communities were visualized using R software. Venn diagrams were plotted using the VennDiagram package in R. Significant differences in the microbiota structure between the two groups were evaluated by nonparametric analysis of similarity (ANOSIM) using the vegan package in R. The specific characterization of the gut microbiota that differentiate among the groups was analyzed using a linear discriminant analysis (LDA) effect size (LEfSe) algorithm (Segata et al., 2011). The parameters set with default p-value, α = 0.05, and an LDA score of 4.0 with LEfS. The relative abundances of OTUs between the GD group and the healthy controls were compared using Statistical Analysis of Metagenomic Profiles (STAMP) software (v2.1.3), Welch’s *t*-test (*p < *0.05), and Benjamini–Hochberg False Discovery Rate (FDR) method (*q < *0.05) as a multiple test correction (Parks et al., 2014). The correlation analysis between clinical parameters and different microbiota was performed using Spearman rank correlation, where a *p*-value < 0.05 was considered statistically significant. Random forest algorithm was used to evaluate the prediction accuracy of the gut microbiota of GD patients and healthy controls. The receiving operational curve (ROC) was analyzed and the area under curve (AUC) using ten-fold cross-validation was calculated. Anthropometric and clinical characteristics were analyzed using SPSS version 18.0 (SPSS Inc., Chicago, USA).

## Results

### Study Population

Fifty-five subjects were enrolled into the GD group and 48 volunteers were enrolled into the healthy control group. The demographic details of the subjects are summarized in **Table 1**. As expected, the GD subjects had a higher FT4 (*p* = 1.98 × 10^-5^) and TPOAb levels (*p* = 1.26 × 10^-10^) compared to the control subjects. The GD subjects also had lower TSH (*p* = 3.73 × 10^-11^). There were no significant differences in age, sex, or body mass index (BMI) (*p* > 0.05).

**Table 1:** Clinical and demographic characteristics of GD patients and healthy controls.

Parameters	GD (N=55)	Control (N=48)	*p*-value
Sex (M/F)	20/35	18/30	0.905
Age (years)	45.09 ± 12.08	42.60 ± 9.78	0.258
BMI (kg/m^2^)	23.84 ± 4.20	23.27 ± 3.41	0.459
FT4 (ng/mL)	2.25 ± 1.58	1.22 ± 0.14	1.98x10^-5^
TSH (mIU/mL)	0.40 ± 0.83	1.48 ± 0.60	3.73x10^-11^
TPOAb (IU/mL), (P/N)	55/0	0/48	1.26x10^-10^

GD, Graves’ disease; M/F, male/female; BMI, body mass index; FT4, free thyroxine; TSH, thyrotropin; TPOAb, thyroperoxidase antibody; P/N, positive/negative ratio. Values are expressed as mean ± standard deviation.

### Diversity Indices and Operational Taxonomic Units (OTUs) Assays of the Gut Microbiota

**Figure 1:**
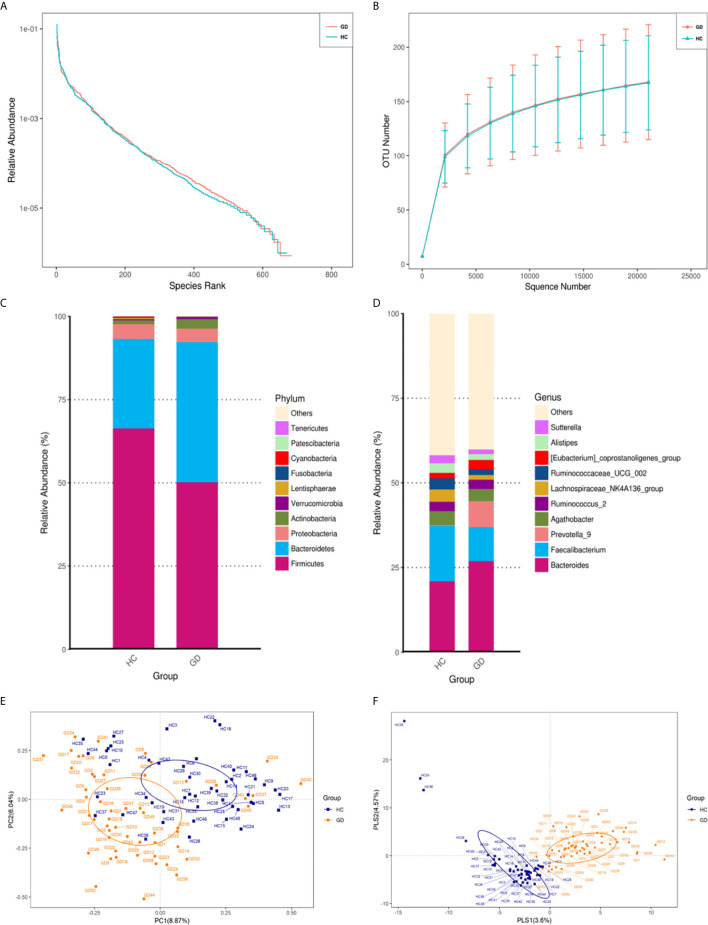
The gut microbiota of GD patients differs from that of healthy controls. Rank-abundance curves were used to explain species richness and evenness **(A)**. Rarefaction curves were used to evaluate the species richness. The bacterial communities of the healthy controls (HC) and GD patients exhibited similar patterns **(B)**. The relative abundances of the top 10 microbial phyla and genera are represented. Only phyla and genera present at relative abundances >1% are shown. Taxa with lower abundances are grouped as “other” **(C, D)**. Principal coordinates analysis (PCOA) based on the distance matrix of Bray-Curtis dissimilarity at the OTU level showed that the gut microbiota of GD patients was separated clearly from those of healthy controls **(E)**. The PLS-DA (Partial Least Squares Discriminant Analysis) showed a significant separation between GD and healthy controls **(F)**.

A total of 11,718,443 usable sequences were obtained from 103 samples using the Illumina MiSeq platform. From these, 5,783,276 high-quality sequences were selected, with an average of 56,158 sequences per barcode sample. Specifically, 671 OTUs in the healthy controls and 684 OTUs in the GD group were delineated at a 97% similarity level. The diversity of the gut microbiota observed in each group is shown in **Table 2**, and detailed characteristics of each sample are shown in **Supplementary Table S1**. The values of Good’s coverage for the two groups were more than 99%, indicating that the current sequencing depth was sufficient to saturate the bacterial diversity of the gut microbiota. The bacterial richness estimates according to ACE and Chao were not significantly different. Analysis of bacterial community richness and evenness with Shannon and Simpson indices showed that the microbiota diversity of GD group, as a whole, was similar to that of the healthy controls. Although both the Shannon and Simpson indices were higher in the GD group, the differences were not statistically significant (*p* > 0.05). Based on the results of the OTU analysis, the rank-abundance curves showed a similar pattern of bacterial communities in the two groups (**Figure 1A**). Rarefaction curve analysis also indicated that the species richness of the GD group exhibited a tendency similar to that of the healthy controls (**Figure 1B**). To better characterize the shared richness within the two groups, the unique and overlapping OTU data were presented in a Venn diagram. These diagrams showed that 589/766 OTUs were shared between the two groups. Thus, 95 and 82 unique OTUs were identified in the GD group and healthy controls, respectively (**Supplementary Figure S1**).

**Table 2:** Phylotype coverage and diversity estimation of the 16S rRNA gene libraries at 97% similarity from the MiSeq analysis.

Group	No. ofreads	No. of OTUs^a^	Good’s (%)^b^	Richness estimators				Diversity index	
				ACE mean	95% C.I.	Chao1 mean	95% C.I.	Shannon mean	Simpson mean
Control	3,570,066	671	0.9985	196.89	182.4568-214.8318	198.05	184.2835-217.6224	4.5097	0.889637
GD	2,213,210	684	0.9985	198.98	188.8540-217.0350	203.01	193.0136-222.4710	4.6725	0.909303

a The operational taxonomic units (OTUs) were defined at 97% similarity level.

b The coverage percentage (Good’s), richness estimators (ACE and Chao1), and diversity indices (Shannon and Simpson) were calculated using Good’s method and the QIIME pipeline (v1.9.1), respectively. C.I., confidence interval.

C.I., confidence interval.

### Differences in Gut Microbiota Composition Between GD and Healthy Controls

To further analyze the composition of the gut microbiota in the different experimental groups, taxonomy from phylum to genus level was assigned to each OTU (**Supplementary Figure S2**). As a result, the relative abundance of the phyla *Bacteroidetes* and *Actinobacteria* was found to increase, while *Firmicutes* decreased in the GD group compared to the healthy controls (**Figure 1C**). In addition, at the genus level, the abundances of *Bacteroides* and *Prevotella_9* were found to be significantly higher, while *Faecalibacterium* and *Lachnospiraceae_NK4A136_group* were slightly lower in the GD group compared to the healthy controls (**Figure 1D**). To measure similarities between the microbial communities, beta diversity analysis was performed using principal coordinate analysis (PCoA) and partial least squares-discriminant analysis (PLS-DA). Despite significant inter-individual variations, the gut microbiota from GD group were clearly different from those of the healthy controls (**Figure 1E**). A clear separation between groups was also observed in PLS-DA (**Figure 1F**). The results of analysis of similarities (ANOSIM) indicated that the structure of the gut microbiota significantly differed between the two groups (ANOSIM, r = 0.158, *p* < 0.001). This comparison revealed that the bacterial diversity of the gut microbiota in the GD group was similar to that observed in the healthy controls. However, the overall community structure was distinctive between the two sample groups.

**Figure 2:**
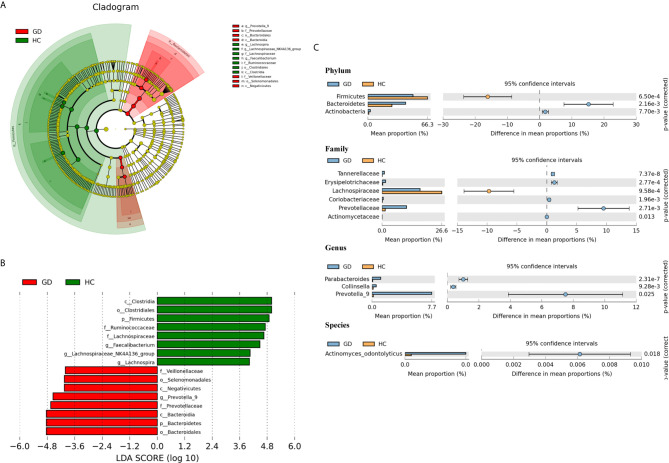
Taxonomic cladogram plotted from linear discriminant analysis (LDA) effect size (LEfSe) analysis and statistical analysis of metagenomic profiles (STAMP) analysis. Linear discriminant analysis effect size (LEfSe) analysis shows bacterial taxa significantly enriched in the GD (red) or healthy controls (HC, green) groups. Taxonomic cladogram and linear discriminant analysis (LDA) scores show differences among taxa between GD and healthy controls. Only taxa meeting a significant LDA threshold value of >4 are shown **(A, B)**. Differentially abundant taxa from the phylum to genus level were further analyzed by STAMP analysis using Welch’s *t*-test (*p* < 0.05, *q* < 0.05) **(C)**.

To identify the specific bacterial taxa associated with GD patients, the gut microbiota in the healthy controls and GD patients was compared using the LEfSe method. A cladogram representing the structure of the gut microbiota and the predominant bacteria in the healthy controls and GD patients is shown in **Figure 2A**, in which the greatest differences in the taxa between the two communities are displayed. LEfSe analysis revealed 16 discriminative features, including 2 phyla, 3 classes, 3 orders, 4 families, and 4 genera (LDA score >4; **Figure 2B**). Bacteria belonging to the *Bacteroidetes* phylum were enriched in the GD patients, while those in *Firmicutes* were more abundant in the healthy controls. At the family level, the proportion of *Prevotellaceae* and *Veillonellaceae* was increased in GD patients, while that of *Lachnospiraceae* and *Ruminococcaceae* was decreased compared to that in healthy controls. Moreover, the level of *Prevotella_9* was significantly higher in GD patients; however, the levels of *Faecalibacterium*, *Lachnospira*, and *Lachnospiraceae_NK4A136_group* were markedly increased in the healthy controls. Thus, these microbial features may be used as potential biomarkers for distinguishing GD patients from healthy controls. The changes in intestinal microbiota composition from the phylum to the species level were further explored by statistical analysis of metagenomic profiles (STAMP) analysis using Welch’s *t*-test. STAMP analysis yielded 22 statistically significant bacterial taxa (*p* < 0.05, q < 0.05) (**Table 3**). At the level of phyla, *Bacteroidetes* and *Actinobacteria* were significantly more abundant in the gut microbiota of GD patients than in healthy controls, whereas *Firmicutes* was less abundant. At the family level, *Prevotellaceae*, *Erysipelotrichaceae*, *Tannerellaceae*, *Coriobacteriaceae*, and *Actinomycetaceae* were prevalent in GD patients, while the family *Lachnospiraceae* was found to be significantly higher in the healthy controls. At the genus and species levels, the abundance of the genera *Prevotella_9*, *Parabacteroides*, and *Collinsella*, as well as those of the species *Actinomyces_odontolyticus*, was higher in the GD patients than in the healthy controls (**Figure 2C**).

**Table 3:** Significant differences in taxa abundances from phylum to species level between GD patients and healthy controls.

Taxonomic level	Bacterial taxa	GD mean	Control mean	*p*-value	*q*-value
Phylum	*Actinobacteria*	2.88	1.11	<0.01	<0.01
	*Bacteroidetes*	42.1	26.9	<0.01	<0.01
	*Firmicutes*	50.2	66.3	<0.01	<0.01
Class	*Bacteroidia*	42.1	26.9	<0.01	<0.01
	*Clostridia*	43.6	64.1	<0.01	<0.01
	*Coriobacteriia*	0.653	0.18	<0.01	<0.01
	*Erysipelotrichia*	1.56	0.31	<0.01	<0.01
Order	*Actinomycetales*	7.17x10^-3^	7.92x10^-4^	<0.01	<0.01
	*Bacteroidales*	42.1	26.9	<0.01	<0.01
	*Clostridiales*	43.6	64.1	<0.01	<0.01
	*Coriobacteriales*	0.653	0.18	<0.01	<0.01
	*Erysipelotrichales*	1.56	0.31	<0.01	<0.01
Family	*Actinomycetaceae*	7.17x10^-3^	7.92x10^-4^	<0.01	0.013
	*Coriobacteriaceae*	0.535	0.142	<0.01	<0.01
	*Erysipelotrichaceae*	1.56	0.31	<0.01	<0.01
	*Lachnospiraceae*	16.9	26.6	<0.01	<0.01
	*Prevotellaceae*	10.9	1.42	<0.01	<0.01
	*Tannerellaceae*	1.1	0.085	<0.01	<0.01
Genus	*Collinsella*	0.523	0.142	<0.01	<0.01
	*Parabacteroides*	1.1	0.085	<0.01	<0.01
	*Prevotella_9*	7.67	0.166	<0.01	0.024
Species	*Actinomyces_odontolyticus*	6.83x10^-3^	6.93x10^-4^	<0.01	0.017

Significant differences between groups are shown as p < 0.05 and q < 0.05, according to Welch’s t-test.

### Correlation Between Clinical Parameters and the Gut Microbiota

**Figure 3:**
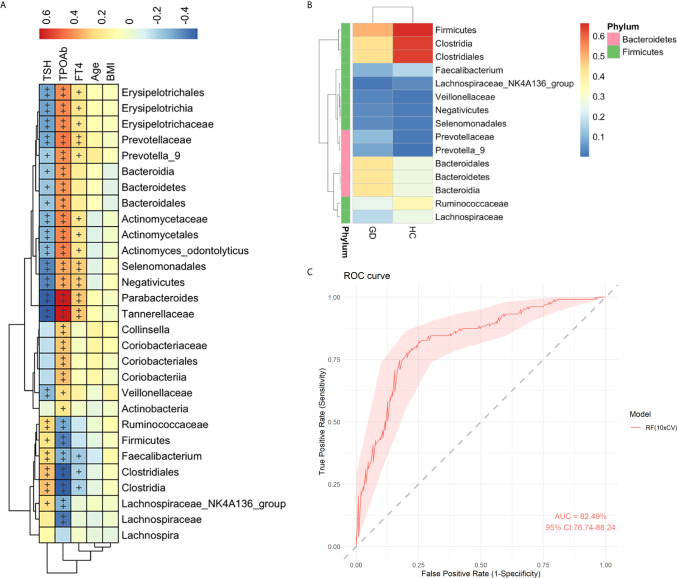
Spearman correlation analysis of the gut microbiota with clinical parameters. Heatmap showing the relationships of altered 29 gut microbiota and 5 clinical parameters in healthy controls and GD patients. Color intensity indicates the magnitude of correlation. Red = positive correlation; blue = negative correlation. ^+^p < 0.05, ^++^p < 0.01. BMI, body mass index; FT4, free thyroxine; TSH, thyrotropin; TPOAb, thyroperoxidase antibody **(A)**. Heatmap analyses of the relative abundant of top 15 taxa from 29 gut microbiota. The heatmap plot depicts the relative abundance of each bacterial taxa (vertical axis) within each group (horizontal axis). The color of the spot in the right panel corresponds to the relative values of the taxa in each group **(B)**. Random forest analysis was used to classify GD patients and healthy controls based on the top 15 most abundant taxa. The performance of the random forest analysis was assessed with the AUC of the ROC curve. The light pink area indicates the 95% confidence interval **(C)**.

To explore the association between the clinical parameters and gut microbiota, a Spearman’s correlation analysis was conducted among five clinical parameters (TSH, TPOAb, FT4, age, and BMI) with 29 bacterial taxa, which were differentially abundant between the GD group and healthy controls in the LEfSe and STAMP analyses (**Figure 2**). The abundance levels of 16 GD-enriched taxa were positively correlated with TPOAb but negatively correlated with TSH, while six taxa enriched in the healthy controls showed contrasting correlations (*p* < 0.05) (**Figure 3A**). Among these, 12 out of 16 GD-enriched taxa were also positively correlated with FT4, while three of the six control-enriched taxa were also negatively correlated with FT4 (*p* < 0.05). However, there were no significant correlations between the gut microbiota and other environmental factors, including age and BMI (*p* > 0.05). A heatmap was generated based on the 15 most abundant taxa from the 29 different taxa. Hierarchical clustering showed a clear separation between the GD group and the healthy controls (**Figure 3B**). Further, to evaluate the predictive power of these taxa in predicting the GD status, the random forest analysis was used and an AUC value of 0.825 was achieved (**Figure 3C**), indicating that these microbial features may be used as potential biomarkers for distinguishing GD patients from healthy controls.

## Discussion

In this study, the gut microbiota of GD patients was compared with those of age- and sex-matched healthy subjects using 16S rRNA sequencing technologies. Patients with GD were found to have an altered gut microbiota composition compared to the healthy controls. In fact, differentially abundant bacterial taxa responsible for the differences between the two groups were identified. In addition, Spearman correlation analysis confirmed that several taxa were significantly correlated with clinical parameters. Among these, the top 15 most abundant taxa discriminated GD patients with high accuracy (AUC = 0.825).

Fecal samples from GD patients were found to show a slight increase in microbial diversity and richness compared to those of the healthy controls, although the differences were not statistically significant (**Table 2**). Previous studies have observed bacterial diversity in hyperthyroid and hypothyroid patients, which may be associated with bacterial overgrowth in the intestinal tract (Lauritano et al., 2007; Zhou et al., 2014). Recently, increased microbial diversity has also been reported in patients with Hashimoto’s thyroiditis and thyroid carcinoma (Zhao et al., 2018; Feng et al., 2019).

PCoA and PLS-DA analyses demonstrated that the microbial compositions of GD patients and healthy controls were divided into two separate groups; the GD group exhibited a relatively high homology, indicating that GD patients may share common microbiota features (**Figures 1E, F**). In our study, GD patients had significantly higher levels of *Bacteroidetes* and *Actinobacteria* and lower levels of *Firmicutes* compared to the healthy controls (**Figure 2** and **Table 3**). These findings are consistent with those of previous studies in patients with GD and Graves’ orbitopathy (Shi et al., 2019; Jiang et al., 2021). However, in other studies of patients with Hashimoto’s thyroiditis or thyroid cancer, *Firmicutes* was found to be more abundant and *Bacteroidetes* less abundant (Zhao et al., 2018; Feng et al., 2019). The ratio of *Firmicutes* to *Bacteroidetes* has been considered as an index of the health of the gut microbiota and has been correlated with obesity (Mariat et al., 2009; Tilg and Kaser, 2011). However, a recent meta-analysis suggested that the way in which the gut microbiota modulates obesity is more complex than a simple imbalance in these commensal phyla.

In addition, our study showed that the fecal samples of the GD patients were rich in members of the species *Actinomyces_odontolyticus*, the genus *Collinsella*, *Parabacteroides*, and *Prevotella_9*, as well as of the family *Veillonellaceae* (**Figures 2B, C**). *Actinomyces odontolyticus* is normally present in the oral cavity and gastrointestinal tract of healthy humans. This species has been shown to be involved in the formation of biofilms on teeth (Liljemark et al., 1993), and has been associated with several human diseases, including actinomycosis, periodontitis, and colorectal cancer (Ximenez-Fyvie et al., 1999; Riegert-Johnson et al., 2002; Yachida et al., 2019). Although its role in the pathogenesis of inflammatory bowel disease has not been demonstrated, it may change the enteric environment and immune factors and aggravate injuries caused by inflammation (Li et al., 2018). The genus *Collinsella* within the family *Coriobacteriaceae* has been previously linked to rheumatoid arthritis and has showed a strong correlation with high levels of alpha-aminoadipic acid and asparagine, as well as the production of the proinflammatory cytokine IL-17A (Chen et al., 2016). *Parabacteroides* can be considered an anti-inflammatory symbiont (Kverka et al., 2011; Hiippala et al., 2020), increased levels of which have been observed in patients with active Behçet’s disease (Ye et al., 2018). *Prevotella* is a known producer of propionate, which can induce the differentiation of regulatory T cells and suppress Th17 polarization (Li et al., 2016). The increased abundance of *Prevotella* has been associated with GD, rheumatoid arthritis and systemic lupus erythematosus (De Aquino et al., 2014; Mendonca et al., 2019; Yan et al., 2020). It may also influence the efficacy of drug therapy for GD (Yan et al., 2020). Our study found significantly high levels of *Prevotella_9* in GD patients, which is consistent with previous reports in GD patients (Ishaq et al., 2018), while decreased levels of *Prevotella_9* have been found in HT patients (Zhao et al., 2018). Besides, our study showed that the abundance of the family *Veillonellaceae* in patients with GD was increased, which is consistent with the previous studies (Yan et al., 2020; Chen et al., 2021). The *Veillonella* genus is normal flora of the mouth, gastrointestinal tract, and vagina. Recent studies have shown that *Veillonella* species were enriched in the fecal microbiota of primary sclerosing cholangitis patients (Little et al., 2020) and may contribute to the immune system development during early childhood (Poppleton et al., 2017).

In addition, our data showed significantly lower levels of the genus *Faecalibacterium*, *Lachnospiraceae NK4A136 group*, and *Lachnospira* in GD patients compared to the healthy controls (**Figures 2B, C**). *Lachnospiraceae NK4A136 group* and *Lachnospira* belong to the family *Lachnospiraceae*, which can digest carbohydrates to produce butyrate, and which has been reported to have potent anti-inflammatory effects (Flint et al., 2012). Previous studies have reported a decrease in the abundance of the genus *Lachnospira* in patients with chronic kidney disease and childhood asthma (Stiemsma et al., 2016; Lun et al., 2019). The relative abundance of *Lachnospiraceae NK4A136 group* has been shown to decrease in children with cystic fibrosis (Coffey et al., 2019). The decrease in *Faecalibacterium* has been linked with GD patients (Ishaq et al., 2018; Zhao et al., 2018; Cornejo-Pareja et al., 2020) and other autoimmune diseases like multiple sclerosis and Crohn’s disease (Thorkildsen et al., 2013; Cantarel et al., 2015). A previous research showed that *Faecalibacterium* was a protective factor, owing to its negative correlation with thyroid stimulating immunoglobulin antibody related to GD patients (Cornejo-Pareja et al., 2020).

Moreover, in addition to investigating alterations of the gut microbiota, we further investigated the relationship between gut microbiota and clinical parameters. TSH, TPOAb, and FT4 were found to be highly correlated with microbiota composition, indicating a close relationship between the gut microbiota and GD (**Figure 3A**). Hierarchical clustering based on the relative abundance of the top 15 taxa from 29 gut microbiota also showed a clear separation between the two groups (**Figure 3B**), and an AUC value of 0.825 was achieved (**Figure 3C**), further indicating that some gut microbiota may be associated with GD. The potential role of gut microbiota in the development of GD warrants further investigation.

In summary, we demonstrated that GD patients, compared to healthy controls, exhibited gut microbiota dysbiosis characterized by unchanged overall bacterial richness and diversity, but an altered taxonomic composition. Several specific differential bacterial taxa were found to be significantly correlated with GD. Nevertheless, this study has several limitations. First, this was a single-center, cross-sectional study involving a limited number of patients. Second, whether the altered gut microbiota is a cause or a consequence of GD development will need to be confirmed. Lastly, gut microbiota studies using 16S rRNA sequencing have yet to provide a detailed list of functional genes and metabolic pathways for the study of host‒gut microbiome interactions. Therefore, further multicenter studies with larger samples using metagenomics and metabolomics integrative analyses are needed to improve our understanding of the interactions between gut microbes, their metabolites, and host cells.

## Data Availability Statement

The datasets presented in this study can be found in online repositories. The names of the repository/repositories and accession number(s) can be found below: https://www.ncbi.nlm.nih.gov/, PRJNA695309.

## Ethics Statement

The studies involving human participants were reviewed and approved by Institutional Review Board and Ethics Committee of Chang Gung Memorial Hospital (IRB 201701806B0C502). The patients/participants provided their written informed consent to participate in this study.

## Author Contributions

S-CC, S-FL, and J-JL designed the experiments. S-FL and S-TC collected the samples and clinical data. P-YC and F-SL helped supervise the project. Y-MY performed the statistical analyses. S-CC and J-JL wrote the manuscript. All authors discussed the results and commented on the manuscript. All authors contributed to the article and approved the submitted version.

## Funding

This work was supported by grants from the Chang Gung Memorial Hospital (CMRPG3F1693).

## Conflict of Interest

The authors declare that the research was conducted in the absence of any commercial or financial relationships that could be construed as a potential conflict of interest.

## Supplementary Material

Supplementary Table 1 Comparison of phylotype coverage and diversity estimation of the 16 rRNA gene libraries at 97% similarity from the sequencing analysis. GD, Graves’ disease; HC, healthy controls.

Supplementary Figure 1 Venn diagram illustrating the overlap of OTUs in the gut microbiota among the two groups

Supplementary Figure 2 Composition analysis of the gut microbiota at different taxonomic levels among the two groups: (A) phylum, (B) class, (C) order, (D) family, (E) genus. Only the top 10 most abundant OTUs are displayed.

## References

[B1] Alkanani A. K. Hara N. Gottlieb P. A. Ir D. Robertson C. E. Wagner B. D.. (2015). Alterations in Intestinal Microbiota Correlate With Susceptibility to Type 1 Diabetes. Diabetes 64, 3510–3520. 10.2337/db14-1847 26068542 PMC4587635

[B2] Cantarel B. L. Waubant E. Chehoud C. Kuczynski J. Desantis T. Z. Warrington J.. (2015). Gut Microbiota in Multiple Sclerosis: Possible Influence of Immunomodulators. J. Investig. Med. 63, 729–734. 10.1097/JIM.0000000000000192 PMC4439263 25775034

[B3] Caporaso J. G. Kuczynski J. Stombaugh J. Bittinger K. Bushman F. D. Costello E. K.. (2010). QIIME Allows Analysis of High-Throughput Community Sequencing Data. Nat. Methods 7, 335–336. 10.1038/nmeth.f.303 20383131 PMC3156573

[B4] Chen J. Wang W. Guo Z. Huang S. Lei H. Zang P.. (2021). Associations Between Gut Microbiota and Thyroidal Function Status in Chinese Patients With Graves’ Disease. J. Endocrinol. Invest. 10.1007/s40618-021-01507-6 33481211

[B5] Chen J. Wright K. Davis J. M. Jeraldo P. Marietta E. V. Murray J.. (2016). An Expansion of Rare Lineage Intestinal Microbes Characterizes Rheumatoid Arthritis. Genome Med. 8, 43. 10.1186/s13073-016-0299-7 27102666 PMC4840970

[B6] Coffey M. J. Nielsen S. Wemheuer B. Kaakoush N. O. Garg M. Needham B.. (2019). Gut Microbiota in Children With Cystic Fibrosis: A Taxonomic and Functional Dysbiosis. Sci. Rep. 9, 18593. 10.1038/s41598-019-55028-7 31819107 PMC6901462

[B7] Cornejo-Pareja I. Ruiz-Limon P. Gomez-Perez A. M. Molina-Vega M. Moreno-Indias I. Tinahones F. J. (2020). Differential Microbial Pattern Description in Subjects With Autoimmune-Based Thyroid Diseases: A Pilot Study. J. Pers. Med. 10 (4), 192. 10.3390/jpm10040192 PMC7712884 33114469

[B8] Correa J. D. Calderaro D. C. Ferreira G. A. Mendonca S. M. Fernandes G. R. Xiao E.. (2017). Subgingival Microbiota Dysbiosis in Systemic Lupus Erythematosus: Association With Periodontal Status. Microbiome 5, 34. 10.1186/s40168-017-0252-z 28320468 PMC5359961

[B9] De Aquino S. G. Abdollahi-Roodsaz S. Koenders M. I. Van De Loo F. A. Pruijn G. J. Marijnissen R. J.. (2014). Periodontal Pathogens Directly Promote Autoimmune Experimental Arthritis by Inducing a TLR2- and IL-1-driven Th17 Response. J. Immunol. 192, 4103–4111. 10.4049/jimmunol.1301970 24683190

[B10] Doubleday A. R. Sippel R. S. (2020). Hyperthyroidism. Gland Surg. 9, 124–135. 10.21037/gs.2019.11.01 32206604 PMC7082267

[B11] Edgar R. C. Haas B. J. Clemente J. C. Quince C. Knight R. (2011). UCHIME Improves Sensitivity and Speed of Chimera Detection. Bioinformatics 27, 2194–2200. 10.1093/bioinformatics/btr381 21700674 PMC3150044

[B12] Ehlers M. Schott M. Allelein S. (2019). Graves’ Disease in Clinical Perspective. Front. Biosci. (Landmark Ed) 24, 35–47. 10.2741/4708 30468646

[B13] Feng J. Zhao F. Sun J. Lin B. Zhao L. Liu Y.. (2019). Alterations in the Gut Microbiota and Metabolite Profiles of Thyroid Carcinoma Patients. Int. J. Cancer 144, 2728–2745. 10.1002/ijc.32007 30565661

[B14] Flint H. J. Scott K. P. Duncan S. H. Louis P. Forano E. (2012). Microbial Degradation of Complex Carbohydrates in the Gut. Gut Microbes 3, 289–306. 10.4161/gmic.19897 22572875 PMC3463488

[B15] Hiippala K. Kainulainen V. Suutarinen M. Heini T. Bowers J. R. Jasso-Selles D.. (2020). Isolation of Anti-Inflammatory and Epithelium Reinforcing Bacteroides and Parabacteroides Spp. From A Healthy Fecal Donor. Nutrients 12 (4), 935. 10.3390/nu12040935 PMC7230855 32230951

[B16] Ishaq H. M. Mohammad I. S. Shahzad M. Ma C. Raza M. A. Wu X.. (2018). Molecular Alteration Analysis of Human Gut Microbial Composition in Graves’ Disease Patients. Int. J. Biol. Sci. 14, 1558–1570. 10.7150/ijbs.24151 30263008 PMC6158725

[B17] Jiang W. Yu X. Kosik R. O. Song Y. Qiao T. Tong J.. (2021). Gut Microbiota may Play a Significant Role in the Pathogenesis of Graves’ Disease. Thyroid. 10.1089/thy.2020.0193 PMC8110022 33234057

[B18] Klindworth A. Pruesse E. Schweer T. Peplies J. Quast C. Horn M.. (2013). Evaluation of General 16S Ribosomal RNA Gene PCR Primers for Classical and Next-Generation Sequencing-Based Diversity Studies. Nucleic Acids Res. 41, e1. 10.1093/nar/gks808 22933715 PMC3592464

[B19] Kornelius E. Yang Y. S. Huang C. N. Wang Y. H. Lo S. C. Lai Y. R.. (2018). The Trends of Hyperthyroidism Treatment in Taiwan: A Nationwide Population-Based Study. Endocr. Pract. 24, 573–579. 10.4158/EP-2017-0266 29688762

[B20] Kverka M. Zakostelska Z. Klimesova K. Sokol D. Hudcovic T. Hrncir T.. (2011). Oral Administration of Parabacteroides Distasonis Antigens Attenuates Experimental Murine Colitis Through Modulation of Immunity and Microbiota Composition. Clin. Exp. Immunol. 163, 250–259. 10.1111/j.1365-2249.2010.04286.x 21087444 PMC3043316

[B21] Lauritano E. C. Bilotta A. L. Gabrielli M. Scarpellini E. Lupascu A. Laginestra A.. (2007). Association Between Hypothyroidism and Small Intestinal Bacterial Overgrowth. J. Clin. Endocrinol. Metab. 92, 4180–4184. 10.1210/jc.2007-0606 17698907

[B22] Lee N. Kim W. U. (2017). Microbiota in T-cell Homeostasis and Inflammatory Diseases. Exp. Mol. Med. 49, e340. 10.1038/emm.2017.36 28546563 PMC5454441

[B23] Li J. Sung C. Y. Lee N. Ni Y. Pihlajamaki J. Panagiotou G.. (2016). Probiotics Modulated Gut Microbiota Suppresses Hepatocellular Carcinoma Growth in Mice. Proc. Natl. Acad. Sci. U. S. A. 113 (9), E1306–E1315. 10.1073/pnas.1518189113 26884164 PMC4780612

[B24] Li J. Li Y. Zhou Y. Wang C. Wu B. Wan J. (2018). Actinomyces and Alimentary Tract Diseases: A Review of Its Biological Functions and Pathology. BioMed. Res. Int. 2018, 3820215. 10.1155/2018/3820215 30225251 PMC6129341

[B25] Liljemark W. F. Bloomquist C. G. Bandt C. L. Pihlstrom B. L. Hinrichs J. E. Wolff L. F. (1993). Comparison of the Distribution of Actinomyces in Dental Plaque on Inserted Enamel and Natural Tooth Surfaces in Periodontal Health and Disease. Oral. Microbiol. Immunol. 8, 5–15. 10.1111/j.1399-302X.1993.tb00536.x 8510984

[B26] Lin L. Zhang J. (2017). Role of Intestinal Microbiota and Metabolites on Gut Homeostasis and Human Diseases. BMC Immunol. 18, 2. 10.1186/s12865-016-0187-3 28061847 PMC5219689

[B27] Little R. Wine E. Kamath B. M. Griffiths A. M. Ricciuto A. (2020). Gut Microbiome in Primary Sclerosing Cholangitis: A Review. World J. Gastroenterol. 26, 2768–2780. 10.3748/wjg.v26.i21.2768 32550753 PMC7284173

[B28] Lun H. Yang W. Zhao S. Jiang M. Xu M. Liu F.. (2019). Altered Gut Microbiota and Microbial Biomarkers Associated With Chronic Kidney Disease. Microbiologyopen 8, e00678. 10.1002/mbo3.678 30088332 PMC6460263

[B29] Magoc T. Salzberg S. L. (2011). FLASH: Fast Length Adjustment of Short Reads to Improve Genome Assemblies. Bioinformatics 27, 2957–2963. 10.1093/bioinformatics/btr507 21903629 PMC3198573

[B30] Mariat D. Firmesse O. Levenez F. Guimaraes V. Sokol H. Dore J.. (2009). The Firmicutes/Bacteroidetes Ratio of the Human Microbiota Changes With Age. BMC Microbiol. 9, 123. 10.1186/1471-2180-9-123 19508720 PMC2702274

[B31] Mendonca S. M. S. Correa J. D. Souza A. F. Travassos D. V. Calderaro D. C. Rocha N. P.. (2019). Immunological Signatures in Saliva of Systemic Lupus Erythematosus Patients: Influence of Periodontal Condition. Clin. Exp. Rheumatol 37, 208–214. 30148445

[B32] Ortega M. A. Fraile-Martinez O. Naya I. Garcia-Honduvilla N. Alvarez-Mon M. Bujan J.. (2020). Type 2 Diabetes Mellitus Associated With Obesity (Diabesity). The Central Role of Gut Microbiota and Its Translational Applications. Nutrients 12 (9), 2749. 10.3390/nu12092749 PMC7551493 32917030

[B33] Parks D. H. Tyson G. W. Hugenholtz P. Beiko R. G. (2014). STAMP: Statistical Analysis of Taxonomic and Functional Profiles. Bioinformatics 30, 3123–3124. 10.1093/bioinformatics/btu494 25061070 PMC4609014

[B34] Picchianti-Diamanti A. Panebianco C. Salemi S. Sorgi M. L. Di Rosa R. Tropea A.. (2018). Analysis of Gut Microbiota in Rheumatoid Arthritis Patients: Disease-Related Dysbiosis and Modifications Induced by Etanercept. Int. J. Mol. Sci. 19 (10), 2938. 10.3390/ijms19102938 PMC6213034 30261687

[B35] Pickard J. M. Zeng M. Y. Caruso R. Nunez G. (2017). Gut Microbiota: Role in Pathogen Colonization, Immune Responses, and Inflammatory Disease. Immunol. Rev. 279, 70–89. 10.1111/imr.12567 28856738 PMC5657496

[B36] Poppleton D. I. Duchateau M. Hourdel V. Matondo M. Flechsler J. Klingl A.. (2017). Outer Membrane Proteome of Veillonella Parvula: A Diderm Firmicute of the Human Microbiome. Front. Microbiol. 8, 1215. 10.3389/fmicb.2017.01215 28713344 PMC5491611

[B37] Quast C. Pruesse E. Yilmaz P. Gerken J. Schweer T. Yarza P.. (2013). The SILVA Ribosomal RNA Gene Database Project: Improved Data Processing and Web-Based Tools. Nucleic Acids Res. 41 (Database issue), D590–D596. 10.1093/nar/gks1219 23193283 PMC3531112

[B38] Riegert-Johnson D. L. Sandhu N. Rajkumar S. V. Patel R. (2002). Thrombotic Thrombocytopenic Purpura Associated With a Hepatic Abscess Due to Actinomyces Turicensis. Clin. Infect. Dis. 35, 636–637. 10.1086/342327 12173147

[B39] Segata N. Izard J. Waldron L. Gevers D. Miropolsky L. Garrett W. S.. (2011). Metagenomic Biomarker Discovery and Explanation. Genome Biol. 12, R60. 10.1186/gb-2011-12-6-r60 21702898 PMC3218848

[B40] Shi T. T. Xin Z. Hua L. Zhao R. X. Yang Y. L. Wang H.. (2019). Alterations in the Intestinal Microbiota of Patients With Severe and Active Graves’ Orbitopathy: A Cross-Sectional Study. J. Endocrinol. Invest. 42, 967–978. 10.1007/s40618-019-1010-9 30674011

[B41] Shukla S. K. Singh G. Ahmad S. Pant P. (2018). Infections, Genetic and Environmental Factors in Pathogenesis of Autoimmune Thyroid Diseases. Microb. Pathog. 116, 279–288. 10.1016/j.micpath.2018.01.004 29325864

[B42] Stiemsma L. T. Arrieta M. C. Dimitriu P. A. Cheng J. Thorson L. Lefebvre D. L.. (2016). Shifts in Lachnospira and Clostridium Sp. in the 3-Month Stool Microbiome are Associated With Preschool Age Asthma. Clin. Sci. (Lond) 130, 2199–2207. 10.1042/CS20160349 27634868

[B43] Szilagyi A. (2020). Relationship(s) Between Obesity and Inflammatory Bowel Diseases: Possible Intertwined Pathogenic Mechanisms. Clin. J. Gastroenterol. 13, 139–152. 10.1007/s12328-019-01037-y 31452062 PMC7101293

[B44] Thorkildsen L. T. Nwosu F. C. Avershina E. Ricanek P. Perminow G. Brackmann S.. (2013). Dominant Fecal Microbiota in Newly Diagnosed Untreated Inflammatory Bowel Disease Patients. Gastroenterol. Res. Pract. 2013, 636785. 10.1155/2013/636785 24348539 PMC3855989

[B45] Tilg H. Kaser A. (2011). Gut Microbiome, Obesity, and Metabolic Dysfunction. J. Clin. Invest. 121, 2126–2132. 10.1172/JCI58109 21633181 PMC3104783

[B46] Ximenez-Fyvie L. A. Haffajee A. D. Martin L. Tanner A. Macuch P. Socransky S. S. (1999). Identification of Oral Actinomyces Species Using DNA Probes. Oral. Microbiol. Immunol. 14, 257–265. 10.1034/j.1399-302X.1999.140410.x 10551171

[B47] Yachida S. Mizutani S. Shiroma H. Shiba S. Nakajima T. Sakamoto T.. (2019). Metagenomic and Metabolomic Analyses Reveal Distinct Stage-Specific Phenotypes of the Gut Microbiota in Colorectal Cancer. Nat. Med. 25, 968–976. 10.1038/s41591-019-0458-7 31171880

[B48] Yan H. X. An W. C. Chen F. An B. Pan Y. Jin J.. (2020). Intestinal Microbiota Changes in Graves’ Disease: A Prospective Clinical Study. Biosci. Rep. 40 (9), BSR20191242. 10.1042/BSR20191242 32820337 10.1042/BSR20191242 PMC7475298

[B49] Ye Z. Zhang N. Wu C. Zhang X. Wang Q. Huang X.. (2018). A Metagenomic Study of the Gut Microbiome in Behcet’s Disease. Microbiome 6, 135. 10.1186/s40168-018-0520-6 30077182 PMC6091101

[B50] Zhao F. Feng J. Li J. Zhao L. Liu Y. Chen H.. (2018). Alterations of the Gut Microbiota in Hashimoto’s Thyroiditis Patients. Thyroid 28, 175–186. 10.1089/thy.2017.0395 29320965

[B51] Zhou L. Li X. Ahmed A. Wu D. Liu L. Qiu J.. (2014). Gut Microbe Analysis Between Hyperthyroid and Healthy Individuals. Curr. Microbiol. 69, 675–680. 10.1007/s00284-014-0640-6 24969306

