# Supplementary material for: Alterations of Gut Microbiota in Patients With Graves’ Disease
Source: Front Cell Infect Microbiol. 2021 May 5;11:663131. doi: 10.3389/fcimb.2021.663131 (PMC8132172; doi:10.3389/fcimb.2021.663131)
Supplement: Supplementary Table 1 — Comparison of phylotype coverage and diversity estimation of the 16 rRNA gene libraries at 97% similarity from the sequencing analysis. GD, Graves’ disease; HC, healthy controls. [file Table_1.docx]

| **Supplementary Table S1.** Comparison of phylotype coverage and diversity estimation of the 16 rRNA gene libraries at 97% similarity from the sequencing analysis | | | | | | | |
| --- | --- | --- | --- | --- | --- | --- | --- |
| Sample | No. of reads | OTUs | Shannon | Simpson | ACE | Chao1 | Good’s coverage |
| GD1 | 28,807 | 170 | 4.107 | 0.8347 | 205.7 | 214.4 | 99.82% |
| GD10 | 42,033 | 177 | 4.880 | 0.9329 | 206.8 | 206.3 | 99.84% |
| GD11 | 39,992 | 161 | 4.777 | 0.9365 | 189.1 | 210.6 | 99.85% |
| GD12 | 31,251 | 287 | 5.471 | 0.9352 | 327.6 | 320.2 | 99.77% |
| GD13 | 38,120 | 160 | 5.360 | 0.9550 | 181.6 | 193.0 | 99.90% |
| GD14 | 45,583 | 273 | 6.161 | 0.9720 | 314.9 | 316.0 | 99.79% |
| GD15 | 75,287 | 119 | 4.237 | 0.8868 | 151.7 | 142.1 | 99.88% |
| GD16 | 35,186 | 139 | 4.386 | 0.9034 | 153.3 | 156.3 | 99.90% |
| GD17 | 37,770 | 145 | 4.648 | 0.9283 | 180.7 | 176.5 | 99.87% |
| GD18 | 30,016 | 157 | 5.138 | 0.9563 | 187.0 | 195.2 | 99.85% |
| GD19 | 34,198 | 238 | 5.119 | 0.9360 | 297.0 | 305.0 | 99.73% |
| GD2 | 39,181 | 178 | 4.995 | 0.9386 | 200.7 | 195.6 | 99.87% |
| GD20 | 37,078 | 109 | 3.896 | 0.8655 | 133.4 | 122.6 | 99.90% |
| GD21 | 33,602 | 154 | 4.788 | 0.9241 | 196.0 | 253.2 | 99.83% |
| GD22 | 30,959 | 161 | 4.919 | 0.9381 | 191.1 | 192.0 | 99.85% |
| GD23 | 33,513 | 122 | 4.063 | 0.8869 | 141.3 | 138.9 | 99.89% |
| GD24 | 36,876 | 244 | 5.179 | 0.9422 | 272.6 | 278.4 | 99.80% |
| GD25 | 39,631 | 265 | 5.413 | 0.9476 | 318.3 | 311.7 | 99.73% |
| GD26 | 116,293 | 158 | 5.087 | 0.9347 | 178.0 | 204.0 | 99.89% |
| GD27 | 21,873 | 124 | 4.950 | 0.9499 | 145.4 | 141.3 | 99.90% |
| GD28 | 36,466 | 119 | 4.537 | 0.9277 | 157.0 | 169.8 | 99.86% |
| GD29 | 90,583 | 121 | 5.031 | 0.9525 | 146.3 | 136.0 | 99.90% |
| GD3 | 46,075 | 195 | 4.838 | 0.9328 | 224.7 | 226.7 | 99.82% |
| GD30 | 41,060 | 169 | 4.640 | 0.9207 | 197.4 | 204.2 | 99.84% |
| GD31 | 37,716 | 164 | 4.165 | 0.8858 | 201.5 | 197.0 | 99.84% |
| GD32 | 35,977 | 133 | 4.272 | 0.8948 | 149.4 | 150.5 | 99.90% |
| GD33 | 30,833 | 184 | 4.993 | 0.9375 | 208.0 | 207.3 | 99.85% |
| GD34 | 34,376 | 133 | 3.569 | 0.7704 | 144.8 | 144.3 | 99.92% |
| GD35 | 38,992 | 140 | 3.932 | 0.8428 | 183.8 | 177.1 | 99.83% |
| GD36 | 35,445 | 175 | 4.885 | 0.9164 | 192.3 | 194.7 | 99.89% |
| GD37 | 103,721 | 213 | 4.722 | 0.9074 | 243.6 | 237.8 | 99.83% |
| GD38 | 32,667 | 163 | 4.860 | 0.9131 | 179.5 | 194.6 | 99.89% |
| GD39 | 35,872 | 230 | 5.429 | 0.9570 | 270.8 | 262.6 | 99.79% |
| GD4 | 36,782 | 112 | 4.220 | 0.8652 | 132.9 | 159.5 | 99.90% |
| GD40 | 68,312 | 167 | 3.878 | 0.8323 | 215.3 | 235.3 | 99.81% |
| GD41 | 43,779 | 130 | 4.161 | 0.8599 | 146.6 | 151.1 | 99.90% |
| GD42 | 34,450 | 202 | 5.296 | 0.9425 | 227.9 | 239.4 | 99.84% |
| GD43 | 32,936 | 182 | 4.677 | 0.9169 | 208.3 | 204.6 | 99.86% |
| GD44 | 40,911 | 238 | 5.016 | 0.8898 | 279.2 | 290.1 | 99.79% |
| GD45 | 49,036 | 246 | 4.906 | 0.9201 | 322.3 | 313.5 | 99.71% |
| GD46 | 30,656 | 119 | 3.971 | 0.8751 | 144.0 | 147.9 | 99.90% |
| GD47 | 35,301 | 149 | 4.607 | 0.9303 | 193.9 | 194.0 | 99.83% |
| GD48 | 43,603 | 197 | 5.040 | 0.9318 | 244.6 | 238.0 | 99.80% |
| GD49 | 31,012 | 162 | 4.064 | 0.8804 | 198.0 | 202.1 | 99.84% |
| GD5 | 38,488 | 159 | 4.630 | 0.8976 | 182.6 | 186.0 | 99.87% |
| GD50 | 31,721 | 125 | 3.324 | 0.7801 | 162.4 | 158.1 | 99.85% |
| GD51 | 41,126 | 163 | 4.098 | 0.8526 | 206.9 | 209.3 | 99.81% |
| GD52 | 34,714 | 201 | 4.859 | 0.9155 | 237.0 | 262.8 | 99.81% |
| GD53 | 36,470 | 126 | 4.850 | 0.9438 | 139.5 | 143.0 | 99.91% |
| GD54 | 37,373 | 102 | 3.922 | 0.8577 | 136.0 | 144.9 | 99.88% |
| GD55 | 38,524 | 143 | 4.310 | 0.8955 | 164.8 | 168.1 | 99.87% |
| GD6 | 40,826 | 148 | 4.707 | 0.9249 | 160.8 | 159.4 | 99.91% |
| GD7 | 38,943 | 170 | 5.220 | 0.9510 | 203.4 | 197.6 | 99.85% |
| GD8 | 54,759 | 155 | 4.958 | 0.9455 | 176.2 | 194.0 | 99.87% |
| GD9 | 29,315 | 156 | 4.823 | 0.9393 | 189.9 | 191.4 | 99.85% |
| HC1 | 92,563 | 155 | 4.502 | 0.8958 | 201.5 | 194.7 | 99.83% |
| HC10 | 69,741 | 199 | 4.287 | 0.8903 | 234.4 | 225.6 | 99.82% |
| HC11 | 47,274 | 150 | 3.191 | 0.6701 | 171.9 | 168.9 | 99.87% |
| HC12 | 64,581 | 218 | 5.129 | 0.9418 | 257.4 | 267.5 | 99.79% |
| HC13 | 62,668 | 217 | 4.720 | 0.9124 | 239.4 | 236.6 | 99.84% |
| HC14 | 76,295 | 187 | 4.945 | 0.9352 | 226.1 | 226.0 | 99.81% |
| HC15 | 69,230 | 192 | 5.003 | 0.9377 | 239.7 | 227.5 | 99.81% |
| HC16 | 48,680 | 92 | 3.119 | 0.7206 | 114.6 | 111.1 | 99.90% |
| HC17 | 73,352 | 176 | 4.482 | 0.8907 | 194.4 | 197.7 | 99.88% |
| HC18 | 39,573 | 120 | 3.206 | 0.7771 | 132.4 | 132.8 | 99.91% |
| HC19 | 84,638 | 166 | 5.680 | 0.9691 | 189.9 | 205.0 | 99.87% |
| HC2 | 57,219 | 169 | 4.741 | 0.9197 | 201.4 | 204.4 | 99.85% |
| HC20 | 27,462 | 149 | 4.609 | 0.9208 | 175.8 | 174.1 | 99.87% |
| HC21 | 82,215 | 157 | 4.541 | 0.9058 | 176.2 | 173.2 | 99.89% |
| HC22 | 76,749 | 121 | 3.246 | 0.7809 | 153.3 | 150.0 | 99.86% |
| HC23 | 68,235 | 161 | 4.538 | 0.9172 | 184.9 | 179.9 | 99.87% |
| HC24 | 83,016 | 294 | 5.787 | 0.9615 | 332.5 | 332.6 | 99.76% |
| HC25 | 127,296 | 152 | 4.288 | 0.8913 | 178.2 | 170.0 | 99.87% |
| HC26 | 94,154 | 303 | 5.867 | 0.9593 | 337.3 | 346.4 | 99.77% |
| HC27 | 101,527 | 92 | 3.095 | 0.6673 | 102.3 | 100.3 | 99.94% |
| HC28 | 70,811 | 255 | 5.633 | 0.9583 | 290.9 | 287.6 | 99.79% |
| HC29 | 58,178 | 104 | 4.751 | 0.9423 | 115.4 | 112.7 | 99.94% |
| HC3 | 103,927 | 125 | 3.736 | 0.8403 | 150.5 | 152.1 | 99.88% |
| HC30 | 84,144 | 113 | 4.266 | 0.9033 | 132.1 | 144.7 | 99.90% |
| HC31 | 88,559 | 134 | 4.272 | 0.8907 | 167.1 | 179.1 | 99.86% |
| HC32 | 29,426 | 212 | 5.017 | 0.9345 | 245.0 | 266.7 | 99.81% |
| HC33 | 82,056 | 234 | 5.446 | 0.9579 | 267.1 | 260.0 | 99.81% |
| HC34 | 130,567 | 149 | 3.496 | 0.7747 | 168.4 | 166.6 | 99.88% |
| HC35 | 90,942 | 125 | 4.468 | 0.9132 | 158.7 | 156.9 | 99.87% |
| HC36 | 210,658 | 147 | 3.676 | 0.8489 | 184.1 | 178.1 | 99.84% |
| HC37 | 61,195 | 103 | 4.597 | 0.9354 | 124.9 | 115.8 | 99.91% |
| HC38 | 41,376 | 199 | 5.189 | 0.9397 | 227.8 | 219.3 | 99.84% |
| HC39 | 64,340 | 164 | 5.233 | 0.9508 | 177.0 | 171.3 | 99.91% |
| HC4 | 82,046 | 201 | 4.853 | 0.9224 | 234.4 | 242.4 | 99.82% |
| HC40 | 45,901 | 235 | 4.952 | 0.9027 | 293.9 | 286.3 | 99.73% |
| HC41 | 92,031 | 240 | 5.392 | 0.9542 | 276.9 | 266.6 | 99.80% |
| HC42 | 50,865 | 132 | 4.512 | 0.9073 | 155.9 | 172.6 | 99.88% |
| HC43 | 113,356 | 182 | 4.274 | 0.8665 | 203.4 | 211.0 | 99.86% |
| HC44 | 33,365 | 105 | 4.201 | 0.9202 | 155.0 | 159.4 | 99.86% |
| HC45 | 69,079 | 134 | 4.090 | 0.8875 | 162.4 | 176.0 | 99.87% |
| HC46 | 89,552 | 272 | 5.919 | 0.9712 | 334.2 | 354.7 | 99.72% |
| HC47 | 80,653 | 139 | 4.330 | 0.8972 | 153.9 | 152.6 | 99.90% |
| HC48 | 111,240 | 122 | 3.780 | 0.8617 | 150.1 | 158.9 | 99.86% |
| HC5 | 64,964 | 219 | 4.726 | 0.9190 | 271.5 | 272.1 | 99.76% |
| HC6 | 58,934 | 139 | 5.127 | 0.9476 | 158.6 | 162.8 | 99.90% |
| HC7 | 64,210 | 82 | 3.170 | 0.7774 | 88.8 | 88.9 | 99.95% |
| HC8 | 61,310 | 173 | 4.769 | 0.9123 | 210.6 | 197.4 | 99.84% |
| HC9 | 69,048 | 116 | 3.612 | 0.8007 | 148.4 | 170.0 | 99.87% |

GD, Graves’ disease; HC, healthy controls.
