# Supplementary material for: Alterations of Gut Microbiota in Patients With Graves’ Disease
Source: Front Cell Infect Microbiol. 2021 May 5;11:663131. doi: 10.3389/fcimb.2021.663131 (PMC8132172; doi:10.3389/fcimb.2021.663131)
Supplement: Supplementary Figure 1 — Venn diagram illustrating the overlap of OTUs in the gut microbiota among the two groups [file Image_1.pdf]

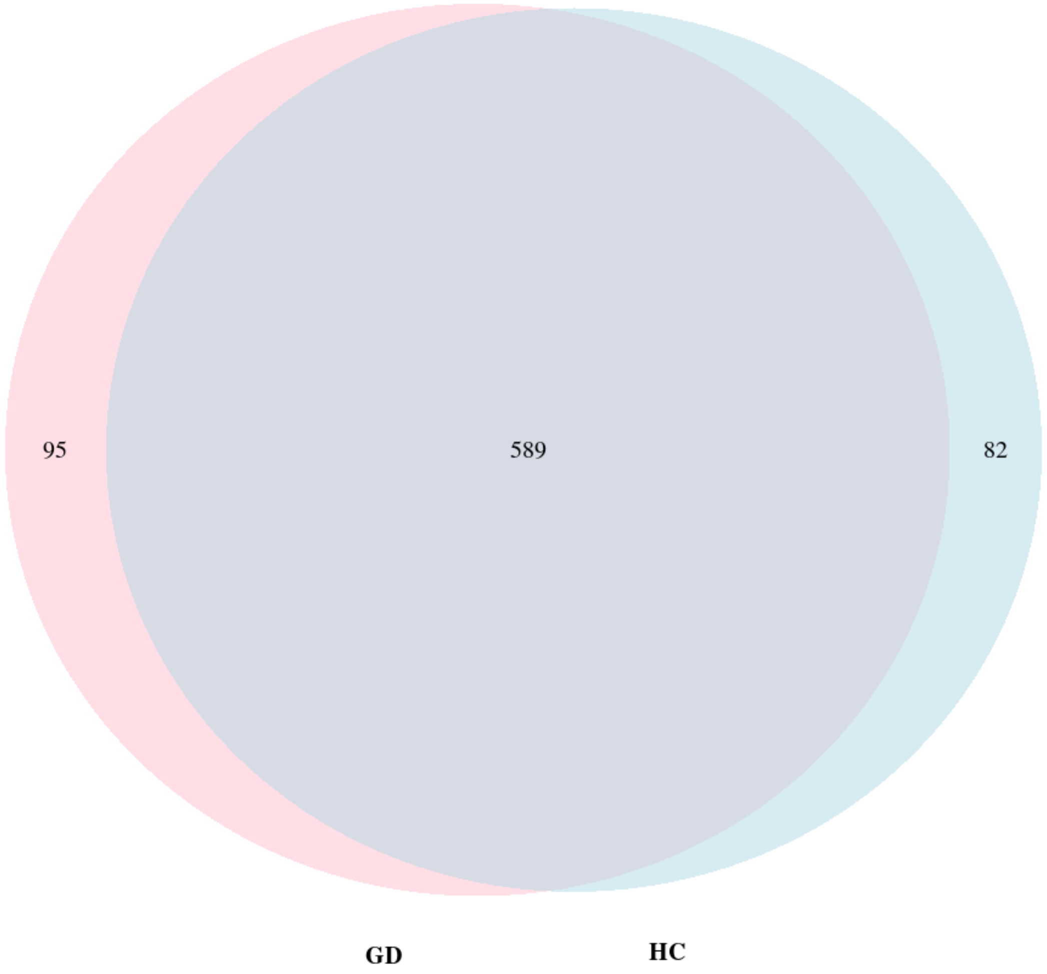

Supplementary Figure S1. Venn diagram illustrating the overlap of OTUs in the gut microbiota among the two groups

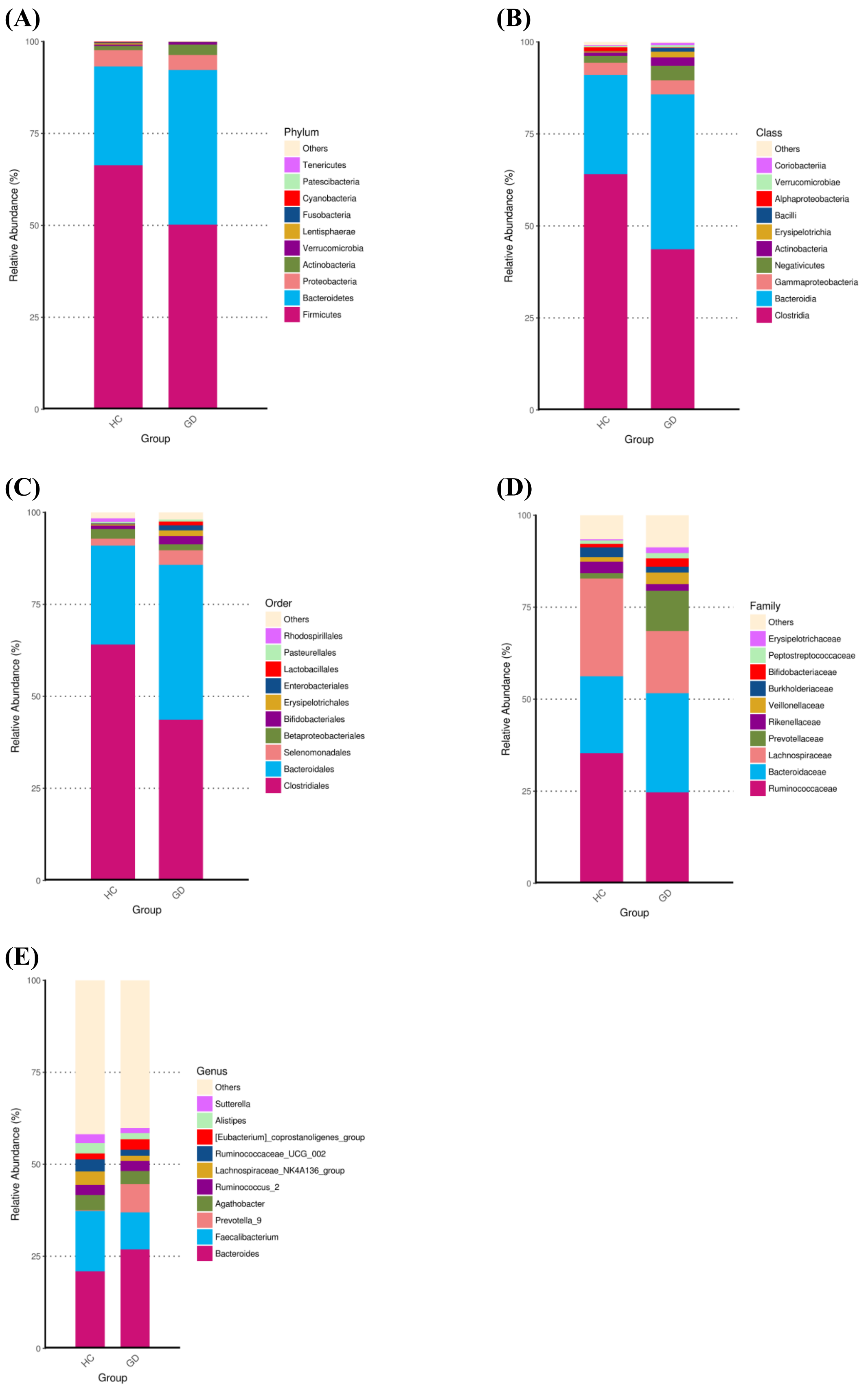
(A)
Relative Abundance (%)
100
75
50
25
0
HC
GD
Group
Phylum
Others
Tenericutes
Patescibacteria
Cyanobacteria
Fusobacteria
Lentisphaerae
Verrucomicrobia
Actinobacteria
Proteobacteria
Bacteroidetes
Firmicutes
(B)
Relative Abundance (%)
Group
Class
Others
Coriobacteriia
Verrucomicrobiae
Alphaproteobacteria
Bacilli
Erysipelotrichia
Actinobacteria
Negativicutes
Gammaproteobacteria
Bacteroidia
Clostridia
(C)
Relative Abundance (%)
Group
Order
Others
Rhodospirillales
Pasteurellales
Lactobacillales
Enterobacteriales
Erysipelotrichales
Bifidobacteriales
Betaproteobacteriales
Selenomonadales
Bacteroidales
Clostridiales
(D)
Relative Abundance (%)
Group
Family
Others
Erysipelotrichaceae
Peptostreptococcaceae
Bifidobacteriaceae
Burkholderiaceae
Veillonellaceae
Rikenellaceae
Prevotellaceae
Lachnospiraceae
Bacteroidaceae
Ruminococcaceae
(E)
Relative Abundance (%)
Group
Genus
Others
Sutterella
Alistipes
[Eubacterium]_coprostanoligenes_group
Ruminococcaceae_UCG_002
Lachnospiraceae_NK4A136_group
Ruminococcus_2
Agathobacter
Prevotella_9
Faecalibacterium
Bacteroides

Supplementary Figure S2. Composition analysis of the gut microbiota at different taxonomic levels among the two groups: (A) phylum, (B) class, (C) order, (D) family, (E) genus. Only the top 10 most abundant OTUs are displayed.
